# Co-design protein sequence and structure in discrete space via generative flow

**DOI:** 10.1093/bioinformatics/btaf248

**Published:** 2025-04-30

**Authors:** Sen Yang, Lingli Ju, Peng Cheng, JiangLin Zhou, Yamin Cai, Dawei Feng

**Affiliations:** Bioinformatics Center of AMMS, Beijing, 100039, China; The 921st Hospital of Chinese PLA, Changsha, 410073, China; Bioinformatics Center of AMMS, Beijing, 100039, China; Bioinformatics Center of AMMS, Beijing, 100039, China; The 921st Hospital of Chinese PLA, Changsha, 410073, China; Computer College, National University of Defense Technology, Changsha, 410073, China

## Abstract

**Motivation:**

Generative models have demonstrated considerable promise in *de novo* protein design. Traditional approaches typically focus on either sequence or structure in isolation, limiting the capacity to explore the intricate sequence–structure landscape and achieve optimal designs. However, joint protein sequence and structure co-design remains a largely underexplored challenge.

**Results:**

We present CoFlow, a discrete model for protein co-design from scratch or given constraints. CoFlow employs a joint discrete flow and integrates a multi-modal protein masked language model to facilitate co-design in the discrete space. Comprehensive experiments demonstrate that CoFlow outperforms previous design methods across multiple evaluation metrics. Notably, CoFlow achieves a consistency approximately eight times higher than that of ESM3 in unconditional generation. Moreover, CoFlow exhibits competitive performance in conditional generation tasks, including motif-scaffolding, protein folding, and inverse folding.

**Availability and implementation:**

The source code of CoFlow, including data preprocessing and model, is available at https://github.com/LtECoD/CoFlow and https://zenodo.org/records/14842367. (DOI: 10.5281/zenodo.14842367).

## 1 Introduction

Over more than 3 billion years of natural evolution, numerous proteins have evolved to perform crucial biological functions in life processes. However, natural proteins are constrained by evolutionary limitations, having adapted to specific ecological and physiological contexts, which often renders them suboptimal for applications such as drug development and enzyme engineering. To overcome these limitations, *de novo* protein design (Huang *et al.* 2016) seeks to create proteins that do not exist in nature but can be tailored for novel applications, including enzymes (Ming *et al.* 2023), antibodies (Joubbi *et al.* 2024), and even molecular switches (Langan *et al.* 2019).

**Figure 1.**
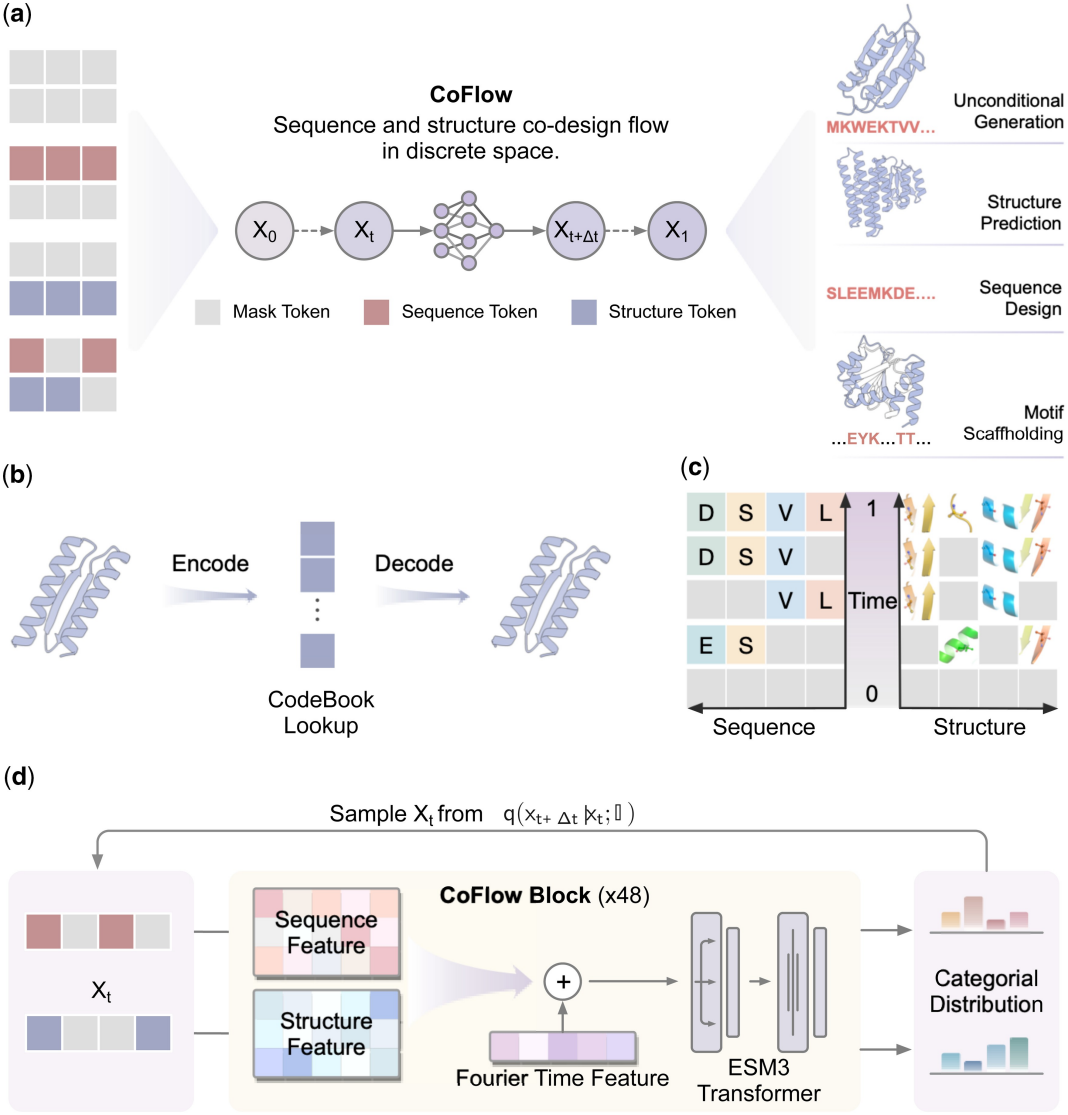
Overview of CoFlow. (a) CoFlow leverages a generative flow to iteratively unmask discrete sequence and structure tokens, allowing for various inputs and supporting both unconditional co-generation and conditional generation tasks, including structure prediction, sequence design, and motif-scaffolding. (b) Discrete protein structure tokens are obtained through the structure VQ-VAE model in ESM3. (c) The joint flow trajectory of sequence and structure is modeled concurrently. (d) The network architecture of CoFlow, which incorporates an additional time feature encoder into ESM3.

Traditional protein design methods emphasize directed evolution (Polizzi and DeGrado 2020) and rational design, typically relying on energy functions and geometric constraints (Leman *et al.* 2020). However, these approaches face limitations in designing structurally and functionally diverse proteins. In recent years, generative artificial intelligence models, such as the language model (Brown *et al.* 2020) and diffusion model (Ho *et al.* 2020), have been introduced for protein design, leading to significant advancements in both protein sequence design (Dauparas *et al.* 2022, Ferruz *et al.* 2022, Madani *et al.* 2023) and structure design (Watson *et al.* 2023, Yim *et al.* 2023, Wang *et al.* 2024) (Supplementary Section S1). As a result, a two-stage paradigm, where the backbone structure and sequence are generated sequentially, has been typically employed to design various proteins. Nevertheless, the information flow in such an approach is unidirectional, being insufficient to capture the intricate sequence–structure landscape that governs protein functionality. Therefore, designing proteins with optimal performance across diverse applications remains challenging. Meanwhile, efforts have been made to co-design protein sequence and structure either sequentially (Anand and Achim 2022, Ingraham *et al.* 2023, Ren *et al.* 2024) or jointly (Campbell *et al.* 2024), yet sequence and structure often remain loosely coupled due to their separate modeling. Recently, ESM3 (Hayes *et al.* 2025), a generative masked language model, was introduced to learn representation and generation across multiple modalities. Specifically, ESM3 employs a VQ-VAE (Van Den Oord *et al.* 2017) to encode protein structure into discrete tokens and decode structure from tokens (Fig. 1b), thus enabling the unified learning of both sequence and structure distributions within a single model. Although ESM3 presents a novel approach to protein co-design, its performance in unconditional generation remains suboptimal (Supplementary Fig. S1). Most structures generated by the released ESM3 exhibit lower pTMs compared to natural proteins.

To overcome the above issue, we propose a novel protein co-design model, CoFlow, which leverages a joint generative flow to co-design protein sequences and backbone in discrete space (Fig. 1a). CoFlow operates within a probabilistic generative framework to model discrete distribution and sample protein instances. It utilizes continuous-time Markov chains (Campbell *et al.* 2024), providing greater flexibility than diffusion models and enabling more diverse and controllable sampling. The generative flow implements linear interpolation (Supplementary Fig. S2) from noise to discrete tokens. To tokenize protein structures and recover three-dimensional coordinates, CoFlow incorporates the structure VQ-VAE from ESM3 (Hayes *et al.* 2025). As a joint generative model, CoFlow integrates two generative flows for sequence and structure, respectively (Fig. 1c). It employs a bidirectional transformer enhanced with layer-wise Fourier time features to model sequence and structure within a unified latent space. The final outputs are two predicted categorical distributions (Fig. 1d). Sampling from CoFlow starts from fully or partially masked tokens, enabling both unconditional generation and various conditional generation tasks.

We conduct comprehensive experiments to evaluate CoFlow against other protein design baselines across various generation tasks. The experimental results show that CoFlow surpasses existing baselines in design consistency for unconditional generation. Notably, CoFlow achieves a consistency that is eight times higher than that of ESM3. In addition, CoFlow demonstrates superior performance in conditional generation tasks, including motif-scaffolding, structure prediction, and sequence design.

## 2 Materials and methods

### 2.1 Preliminary

A protein can be modeled as a linear chain of residues, each associated with an amino acid and three-dimensional atomic coordinates. To convert continuous coordinates into discrete tokens, we utilize the structure VQ-VAE in ESM3 (Hayes *et al.* 2025), which exhibits exceptional capability in encoding and decoding protein structures (Supplementary Section S6). Consequently, we represent a protein as x=(s,r), where $s=s^{1},s^{2},\ldots ,s^{N}$ denotes the sequence tokens (i.e. amino acids), and $r=r^{1},r^{2},\ldots ,r^{N}$ represents backbone structure tokens. Here, the superscript indicates the residue index, and *N* is the total number of residues in the protein.

Protein co-design in discrete space involves generating a protein x, specifically the sequence tokens s and the structure tokens r. Similar to the diffusion model, the generative flow samples x iteratively, from $x_{0}=(s_{0},r_{0})$ to $x_{1}=(s_{1},r_{1})$, where $x_{0}$ represents the initial input and $x_{1}$ denotes the generated sample. Depending on the content of x, the co-design paradigm encompasses a range of tasks (Fig. 1a). If $x_{0}$ is fully masked, the model performs unconditional co-design. If $s_{0}$ is given but $r_{0}$ is masked, the model predicts the folding of the protein. Conversely, if $r_{0}$ is given and $s_{0}$ is masked, the model performs inverse folding. Finally, if a subset of residues is given with their sequence and structure tokens, the model then scaffolds the motif. We denote the sequence mask token as $M_{s}$ and the structure mask as $M_{r}$. Additionally, we use the notation *M* to represent a generic mask when sequence and structure specifications are not distinguished.

### 2.2 The joint flow

The joint discrete generative flow, denoted as $p_{t}$, represents a probability distribution that transitions smoothly from noise to data, where $p_{0}$ is the white noise distribution $p_{\text{noise}}$ and $p_{1}$ is the data distribution $p_{\text{data}}$. Constructing $p_{t}$ directly is non-trivial; thus, we define it through a simplified conditional flow over data points that can be explicitly modeled (Campbell *et al.* 2024). Accordingly, $p_{t}$ is expressed as:


(1) $$p_{t}=\underset{x_{1}∼p_{\mathrm{data}}}{E}p(x_{t}|x_{1})$$

As the protein x comprises sequence s and structure r, we decompose $p(x_{t}|x_{1})$ as:


(2) $$p(x_{t}|x_{1})=p(s_{t}|s_{1})p(r_{t}|r_{1})$$

This decomposition assumes independence between sequence and structure during interpolation. Additionally, we assume residues are also independent. Consequently, for simplicity, residue-level notations such as p(s) and p(r) are used instead of p(s) and p(r) in the following ($p(s_{t})=\prod_{i=1}^{N}p(s_{t}^{i})$ and $p(r_{t})=\prod_{i=1}^{N}p(r_{t}^{i})$):


(3) $$p(x_{t}|x_{1})=p(s_{t}|s_{1})p(r_{t}|r_{1})$$

Using linear interpolation, $p(s_{t}|s_{1})$ is given by:


(4) $$p(s_{t}|s_{1})=tf(s_{1})+(1-t)f(M_{s})$$

where *f* represents the one-hot encoding function. $p(r_{t}|r_{1})$ can be formulated like $p(s_{t}|s_{1})$. Detailed derivations are provided in Supplementary Section S2.

The generative flow aims to sample a trajectory from $x_{0}$ to $x_{1}$ using a generative model parameterized by θ. Specifically, the *t*-step posterior distribution $q(x_{t+\Delta t}|x_{t};\theta )$ can be deduced as (Supplementary Section S3):


(5) $$\{\begin{array}{cc} \frac{\Delta t}{1-t}f_{\theta }+\left(1-\frac{\Delta t}{1-t}\right)f(M) & \text{if}\;x_{t}=M\\ f(x_{t}) & \text{otherwise} \end{array}$$

where $f_{\theta }$ represents the predicted categorical distribution over the token vocabulary, ensuring that the probability of the mask token *M* is zero. Equation (5) implies that if $x_{t}$ is the mask token *M*, $x_{t+\Delta t}$ is sampled from the predicted distribution with a probability of $\frac{\Delta t}{1-t}$; otherwise, $x_{t+\Delta t}$ remains masked as $x_{t}$. To enable transitions between the mask token and other states, we follow Campbell *et al.* (2024) by introducing balanced noise to $q(x_{t+\Delta t}|x_{t};\theta )$. Consequently, $q(x_{t+\Delta t}|x_{t};\theta )$ becomes:


(6) $$\{\begin{array}{cc} \frac{1+\eta t}{1-t}\Delta tf_{\theta }+\left(1-\frac{1+\eta t}{1-t}\Delta t\right)f(M) & \text{if}\;x_{t}=M\\ \left(1-\eta \Delta t)f(x_{t})+\eta \Delta tf(M\right) & \text{otherwise} \end{array}$$

This adjustment introduces a bonus probability of $\frac{\eta t\Delta t}{1-t}$ for unmasking and allows x≠M to transition to *M* with a probability of ηΔt. Note that Equation (6) allows non-masked tokens to revert to masked only before the last sampling step. However, at the last step, this probability becomes zero, ensuring that non-masked tokens do not revert to masked at the final step.

### 2.3 Model

Figure 1d illustrates the overall architecture of the CoFlow model. It takes sequence and structure tokens, along with the time step *t*, as input. The neural network architecture is a transformer with the initial protein representation formed by summing embeddings of sequence and structure. The model’s trunk consists of 48 blocks, each containing a transformer variant module proposed in ESM3 (Hayes *et al.* 2025), augmented by layer-wise Fourier time features. The output of CoFlow includes two distributions for each residue: sequence distribution $f_{\theta }^{s}$ and structure distribution $f_{\theta }^{r}$. During training, a time step *t* is randomly sampled, and the interpolated protein $x_{t}$ is computed according to Equation (2). This interpolated input is used to predict $x_{1}$, with the training objective defined as minimizing the cross-entropy loss. We train CoFlow using proteins ranging in length from 40 to 512 from the MGnify30 (Lin *et al.* 2023) dataset. As a result, CoFlow is well-suited for generating proteins with lengths up to 512. Training details can be found in Supplementary Section S4.

## 3 Results and discussion

### 3.1 Unconditional generation

We first evaluate CoFlow against several strong baselines on the unconditional generation task, which involves sampling amino acid sequence and three-dimensional atom coordinates without any conditional restrictions. Following the methodology in Multiflow (Campbell *et al.* 2024), we assess the generated proteins in terms of consistency, diversity, and novelty. Consistency is measured using scRMSD that quantifies the divergence between the generated backbone structure and the predicted backbone by AlphaFold2 (Jumper *et al.* 2021) from the generated sequence. Diversity is defined as one minus the average pairwise TMScore (Zhang and Skolnick 2004) among generated consistent proteins (scRMSD < 2 Å). A high average pairwise TMScore indicates significant similarity among the generated proteins, leading to low diversity, whereas a lower TMScore suggests higher diversity. Similarly, for each generated protein, novelty is quantified as one minus the highest TMScore between it and proteins in the PDB database. A lower TMScore reflects greater novelty in the generated proteins.

**Figure 2.**
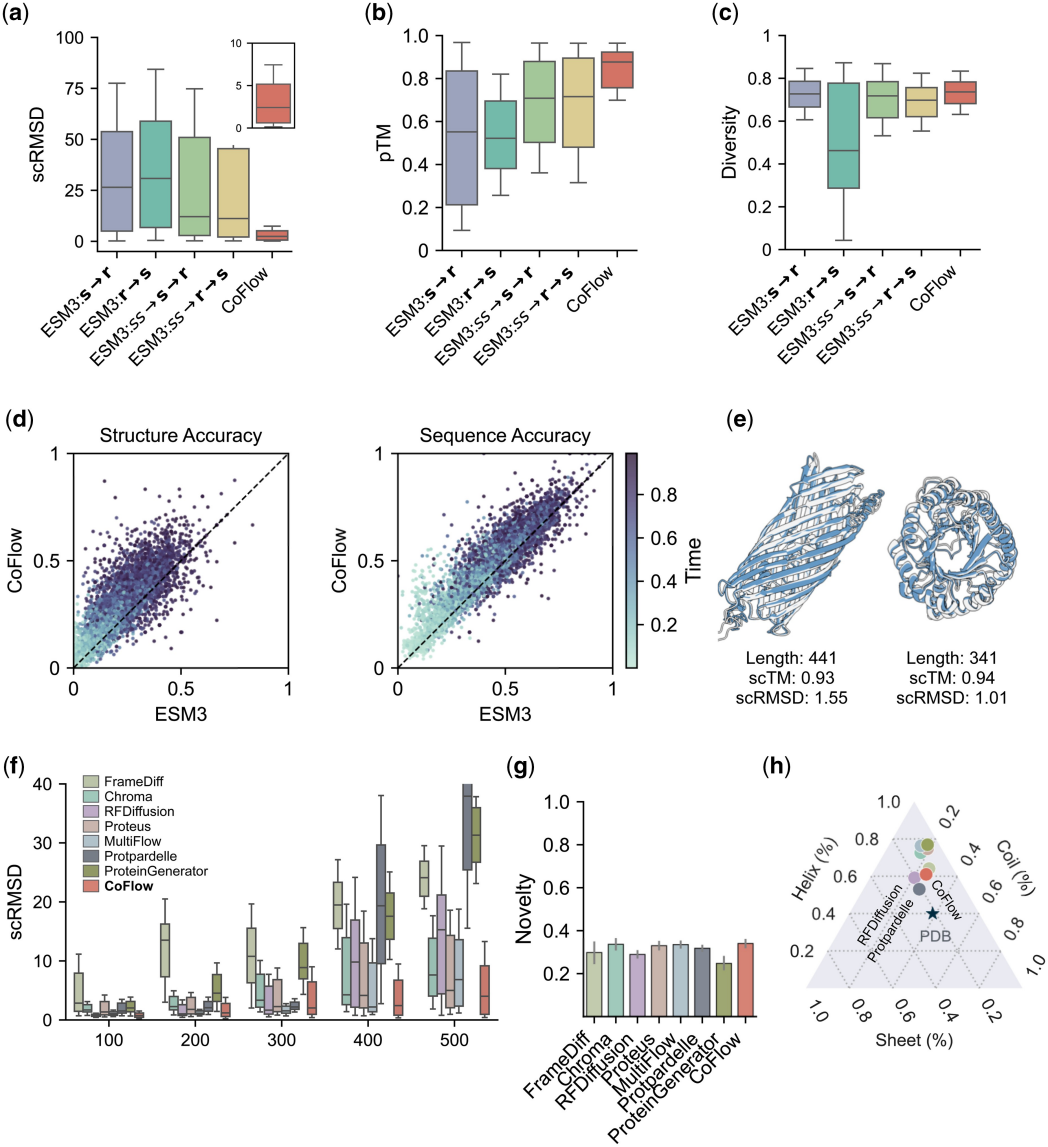
Comparison of unconditional generation. (a–c) CoFlow is compared with ESM3 across various sampling strategies using metrics including scRMSD, pTM, and diversity. (d) The prediction accuracy for structure and sequence tokens is also evaluated in a comparative analysis between CoFlow and ESM3. (e) Illustration of two structures generated by CoFlow alongside their corresponding predictions. (f–h) Additionally, CoFlow is benchmarked against several strong baselines in terms of scRMSD, novelty, and secondary structure distribution.

In Fig. 2a–c, we conduct a comparative evaluation with four ESM3 (our experiments are based on ESM3-open) variants with different sampling strategies (Supplementary Section S5). For each model, 500 proteins of random lengths are sampled. All four models exhibit high average backbone scRMSD values (Fig. 2a), indicating poor consistency between the generated sequences and their corresponding structures. Additionally, the pTM distribution of the ESM3 models demonstrates a large variance (Fig. 2b), suggesting that many of the generated structures are disordered (Supplementary Fig. S1). It indicates a significant limitation of ESM3 in unconditional structure generation. In contrast, CoFlow achieves markedly better performance compared to the four ESM3 models, with higher consistency and pTM scores. The average scRMSD for CoFlow is 3.1 Å, whereas the lowest average scRMSD among the ESM3 models is 24.3 Å (achieved by ESM3: ss→s→r), making CoFlow’s consistency approximately eight times that of ESM3. For pTM, CoFlow demonstrates a significantly higher mean value and lower variance than ESM3, indicating its capability to generate more plausible protein structures. Regarding diversity, CoFlow exhibits a slight superiority over ESM3 (Fig. 2c). Examples of proteins designed unconditionally by CoFlow are presented in Fig. 2e and Supplementary Fig. S4.

We further compare the unmasking accuracy of CoFlow and ESM3 (Fig. 2d). Using a hold-out dataset of 10K proteins, we linearly interpolate sequence and structure tokens over time and task both models with predicting the masked tokens. Overall, the prediction accuracy for structure tokens is lower than that for sequence tokens, which is expected given that the structure vocabulary is larger than the sequence vocabulary, making structure prediction more challenging. Notably, CoFlow demonstrates significantly higher prediction accuracy than ESM3, particularly for structure tokens. This enhanced accuracy in structure prediction reinforces the superior consistency of CoFlow compared to ESM3.

CoFlow is also compared with previous strong baselines, including backbone structure generation models such as FrameDiff (Yim *et al.* 2023), RFDiffusion (Watson *et al.* 2023), and Proteus (Wang *et al.* 2024), as well as protein co-design models like Chroma (Ingraham *et al.* 2023), MultiFlow (Campbell *et al.* 2024), Protpardelle (Chu *et al.* 2024), and ProteinGenerator (Lisanza *et al.* 2024). For each model, we generate 100 proteins with lengths of 100, 200, 300, 400, and 500 residues. For structure design baselines, we additionally use ProteinMPNN (Dauparas *et al.* 2022) to design sequences for the generated backbones. As shown in Fig. 2f, CoFlow exhibits comparable consistency with previous models for proteins shorter than 300 residues. However, it outperforms other baselines in generating proteins of 400 and 500 residues, highlighting its advantage in handling long proteins. In terms of novelty, CoFlow achieves performance comparable to Chroma, Proteus, MultiFlow, Protpardelle, and Proteingenerator (Fig. 2g). Furthermore, we examine the secondary structure distributions of all models in comparison to those of natural proteins from the PDB database. As illustrated in Fig. 2h, the secondary structure distribution of CoFlow closely approximates that of RFDiffusion, but inferior to another co-design method Protpardelle.

### 3.2 Conditional generation

Next, we conduct experiments to evaluate the performance of CoFlow on conditional generation tasks, including motif-scaffolding, folding, and inverse folding. Motif-scaffolding (Trippe *et al.* 2023) involves generating a compatible scaffold while preserving a given motif structure. Folding (Jumper *et al.* 2021) aims to predict the 3D structure of a specified amino acid sequence, whereas inverse folding (Dauparas *et al.* 2022) seeks to reconstruct the amino acid sequence from a given 3D structure.

**Figure 3.**
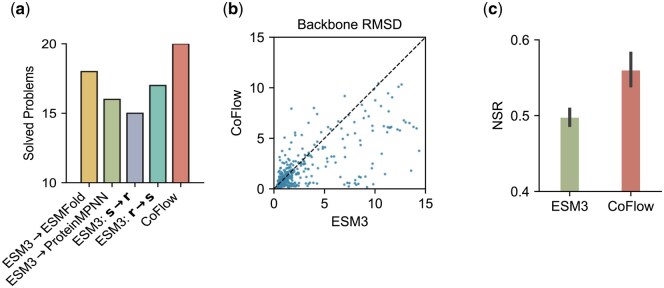
Comparison with ESM3 on conditional generation. (a) Solved problems in the motif-scaffolding task. (b) Backbone RMSD for structure prediction. (c) Recovery rate in the inverse folding task.

For the motif-scaffolding task, we use the dataset curated by Yim *et al.* (2024), which includes 24 specific scaffolding problems. For each problem, we randomly sample lengths that satisfy the given constraints, keeping the sequence and structure tokens of the specified motif unchanged while masking all other tokens. These inputs are then provided to the generative model to sample 100 candidates. A generated sample is considered successful if it meets two criteria: (1) the TMScore between the generated structure and the predicted structure exceeds 0.8, and (2) the predicted motif structure matches the native structure with a backbone RMSD of less than 1 Å. Like the setting in unconditional generation, we compare CoFlow against four models: the first generates sequences using ESM3 and predicts structures with ESMFold (Lin *et al.* 2023); the second generates structures with ESM3 and predicts sequences using ProteinMPNN (Dauparas *et al.* 2022); the last two employ ESM3 to generate sequences and structures in different orders. Figure 3a shows that CoFlow effectively generates reasonable scaffolds for the given functional motifs, successfully solving 20 out of 24 problems and outperforming all ESM3-based models. Additionally, Supplementary Fig. S5 illustrates successful scaffolds for each motif generated by CoFlow.

For the folding and inverse folding tasks, we utilize the test dataset curated by Campbell *et al.* (2024), which consists of 438 monomers excluded from the fine-tuning dataset. We evaluate the performance of ESM3 and CoFlow by comparing the backbone RMSD between predicted and ground truth structures. As shown in Fig. 3b, CoFlow achieves superior performance over ESM3 in a zero-shot setting. For the inverse folding task, we evaluate the average native sequence recovery (NSR) of CoFlow and ESM3. Figure 3c demonstrates that CoFlow achieves an average NSR of 0.56, compared to 0.5 for ESM3, indicating that CoFlow is more effective in generating accurate sequences that fold into the specified structures.

### 3.3 Ablation study

**Figure 4.**
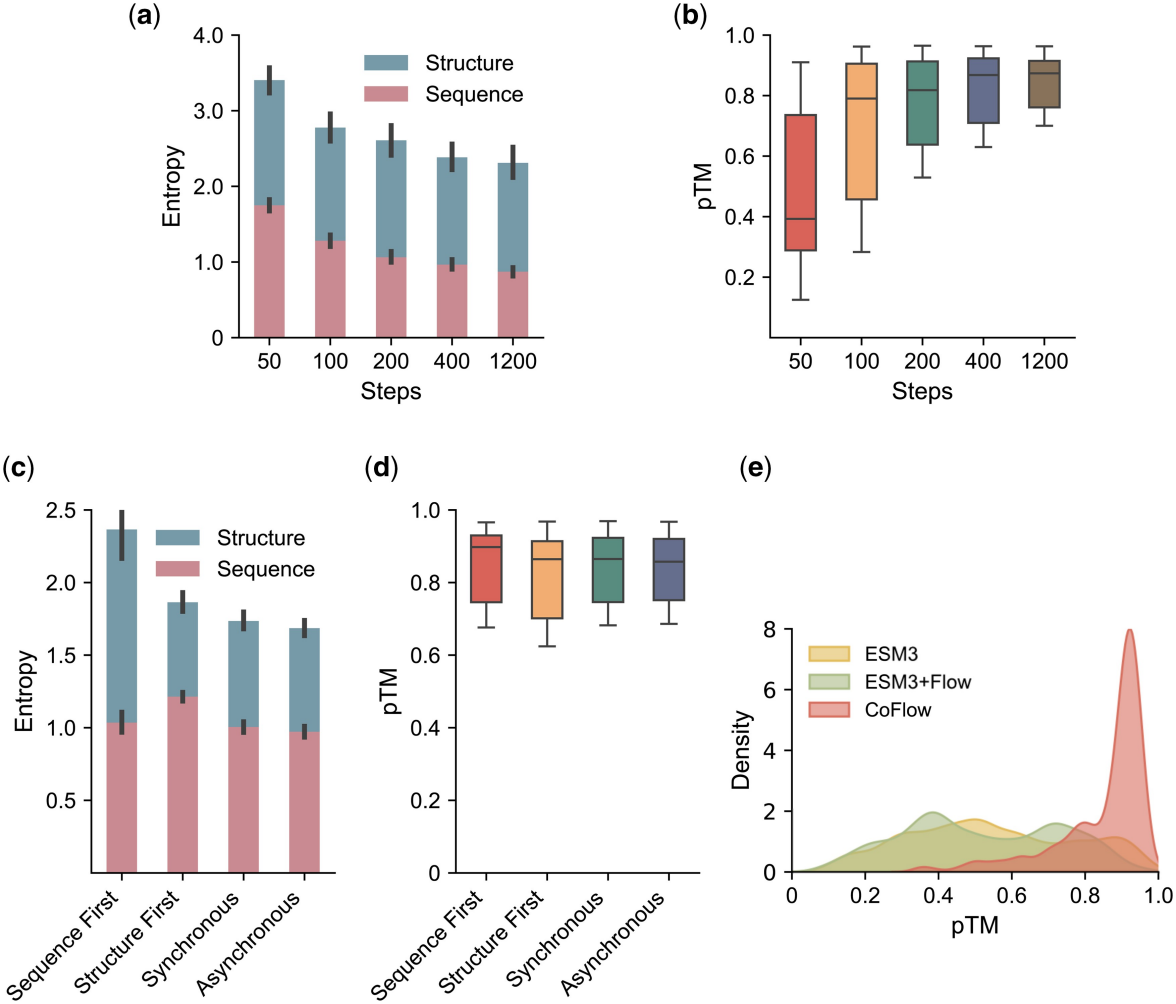
Ablation study. Generation entropy (a) and pTM distribution of generated structures (b) with different sampling steps. Generation entropy (c) and pTM distribution of generated structures (d) with different sampling strategies. (e) pTM distribution of structures generated by ESM3, ESM3 equipped with generative flow, and CoFlow.

We evaluate the impact of sampling steps on protein generation. Specifically, we randomly sample 200 proteins using sampling steps of 50, 100, 200, 400, and 1200 and compare the generation entropy sum of sequence and structure, as well as the pTM score of the generated structure. Figure 4a shows that the entropy gradually decreases as the number of sampling steps increases. Similarly, Fig. 4b illustrates that the average pTM score improves with more sampling steps. These results indicate that increasing the number of sampling steps enables the model to generate proteins with greater confidence. However, the improvement is not linear; the gain diminishes as the number of steps increases. For instance, increasing the sampling steps from 50 to 400 almost doubles the average pTM score, whereas further increasing the steps to 1200 yields only a marginal improvement. Based on this analysis, we set the sampling steps to 400 to achieve a balance between sampling efficiency and generation quality. We also evaluate the entropy of ESM3 across different sampling steps (Supplementary Fig. S6). Its entropy remains nearly constant when the sampling steps exceed 200, converging from a maximum of 2.9 to 2.5. In contrast, CoFlow exhibits a continuous decrease in entropy as the sampling steps increase, dropping from 3.4 to 2.3 (Fig. 4a). This indicates that the gain provided by increasing sampling steps is relatively smaller for ESM3 compared to CoFlow.

We validate the four sampling strategies (Supplementary Section S5) to explore the effect of generation order for sequence and structure tokens. These strategies produce similar pTM scores for the generated structures (Fig. 4d). However, the asynchronous strategy achieves the lowest generation entropy (Fig. 4c). The result is consistent with the intuitive understanding that alternating the generation of sequence and structure tokens allows the model to more effectively capture the complex correlations between them, thereby enhancing generation quality. Consequently, we adopt the asynchronous sampling strategy in unconditional generation.

Since CoFlow shares a similar training objective with ESM3, i.e. predicting masked tokens, we evaluate the effectiveness of directly integrating ESM3 with the flow model. Figure 4 presents the pTM distribution of structures generated by the three models using kernel density estimation. The results indicate that while ESM3 combined with discrete flow performs comparably to its naive counterpart, CoFlow exhibits significant superiority over both. We attribute this performance improvement to two key factors. First, ESM3 is trained with a masking ratio of approximately 25%–30%, whereas CoFlow is trained with a variable masking ratio ranging from 0% to 100%. Consequently, ESM3 struggles when generating from scratch, as it lacks initial token guidance, leading to its underperformance in this comparison. Second, ESM3 does not incorporate a time feature, meaning it lacks an explicit mechanism to track the progression of iterative generation. In contrast, CoFlow integrates time features, allowing it to dynamically adjust its generation process at different stages, thereby facilitating high-quality iterative generation.

## 4 Conclusion

In this article, we propose CoFlow, a discrete flow-based model designed to jointly generate protein sequence and structure. Our experiments demonstrate that CoFlow outperforms ESM3 and other strong baselines across various generation tasks. Despite these promising results, several limitations warrant further investigation. One limitation is that CoFlow does not currently incorporate or generate side-chain structures, which are known to be critical for improving the accuracy and realism of molecular modeling. While this aspect lies beyond the current scope and objectives of our work, we recognize its importance and consider it a key direction for future research to further enhance the precision and biological applicability of CoFlow. Large language models consistently adhere to scaling laws, wherein performance improves with increased model size, given sufficient data and computational resources. Although our model is currently constrained in parameter scale, it has already achieved notable results, suggesting that further scaling of model size or training data could unlock additional potential and significantly enhance performance. Additionally, our work has primarily focused on monomer design and has not yet addressed the design of protein complexes, underscoring a critical direction for future research. Lastly, our evaluation of CoFlow has been limited to *in silico* experiments. Wet-lab validation remains essential for a comprehensive assessment of the model’s practical utility and constitutes a key focus for future studies.

## Supplementary Material

btaf248_Supplementary_Data

## Code availability

Source codes in this study are available at https://github.com/LtECoD/CoFlow and https://zenodo.org/records/14842367.
