# Supplementary material for: Co-design protein sequence and structure in discrete space via generative flow
Source: Bioinformatics. 2025 Apr 30;41(5):btaf248. doi: 10.1093/bioinformatics/btaf248 (PMC12094817; doi:10.1093/bioinformatics/btaf248)
Supplement: btaf248_Supplementary_Data [file btaf248_supplementary_data.pdf]

Supplemental Material

1 Related Work

Protein design has gained significant attention due to its potential to create novel proteins with tailored functions for various applications. Traditional protein design approaches often rely on energy minimization (Leman et al., 2020) and dicreted evaluation (Arnold, 1996), to evaluate and optimize protein structures. However, recent advances in generative artificial intelligence have revolutionized the field. AI-driven methods, such as AlphaFold (Abramson et al., 2024; Jumper et al., 2021) for protein structure prediction, and generative models like language model (Brown et al., 2020; Kenton and Toutanova, 2019) and diffusion model (Ho et al., 2020; Watson et al., 2023), have enabled the generation of high-quality protein sequence or structure, significantly advancing the efficiency and accuracy of protein design.

Protein language models have emerged as powerful tools for understanding and designing proteins by treating amino acid sequences as a ”biological language.” Masked protein language models, such as ESM (Lin et al., 2023a), ProtTrans (Elnaggar et al., 2021), PRoBERTa (Nambiar et al., 2020), and ProteinBERT (Brandes et al., 2022), as well as autoregressive models like ProGen (Madani et al., 2023) and ProtGPT2 (Ferruz et al., 2022), leverage self-supervised learning on large-scale protein sequence datasets to capture evolutionary and structural information. These models encode sequence representations that effectively preserve functional and structural relationships. Recent advancements demonstrate that large protein language models can not only generate novel proteins with desired properties that extend beyond natural ones but also enable a wide range of downstream tasks, including protein structure prediction (Lin et al., 2023b), function annotation (Hayes et al., 2025), antibody infilling (Melnyk et al., 2023), and mutation effect prediction (Cheng et al., 2024).

Recently, the diffusion model has emerged as an advanced approach in protein design. Inspired by their success in image generation (Ramesh et al., 2021; Rombach et al., 2022), diffusion model, that iteratively transforms white noise into targets, is particularly well-suited for navigating the complex solution space of proteins. Methods such as RFDiffusion (Watson et al., 2023) and other diffusion-based frameworks (Liu et al., 2024; Wang et al., 2024; Wang et al.; Wu et al., 2024; Yim et al., 2023) have demonstrated their ability to design diverse, high-quality protein backbones while simultaneously incorporating functional, structural, and even natural language constraints (Ingraham et al., 2023). Unlike traditional generative models, the diffusion model excels at learning continuous and smooth distributions over the protein structure space, being able to conduct fine-grained control over properties such as function and shape. These advantages reveal the potential of diffusion model as a robust and flexible method for *de-novo* protein design. However, diffusion models naturally run in continuous spaces, such as three-dimensional protein coordinates, making their extension to discrete sequence space prone to inefficiencies and

inaccuracies. This limitation has driven the development of discrete flow models (Campbell et al., 2024), which offer an alternative framework for protein sequence generation. Discrete flow models retain the ability to model complex distributions while operating directly on discrete tokens, enabling more efficient and accurate sequence generation. Our method leverages a discrete flow model to design protein sequences and structures jointly.

Protein co-design aims to optimize protein sequences and their corresponding 3D structures simultaneously. While recent models (Campbell et al., 2024; Chu et al., 2024a,b; Lisanza et al., 2024; Ren et al.) have advanced sequence-structure integration, significant challenges persist. Most current co-design approaches rely on a two-stage design strategy, where protein sequence and structure design are treated as independent processes. This sequential workflow often leads to suboptimal results, as it fails to fully account for the intricate interdependence between sequence and structure.

2 Interpolation

We first define a probability transition matrix for interpolation as:

$$Q_\tau = \begin{bmatrix} 1-\tau & & & \tau \\ & 1-\tau & & \tau \\ & & \ddots & \vdots \\ & & & 1 \end{bmatrix} \tag{1}$$

Here, Q is an n-dimensional square matrix, where n represents the vocabulary size. We assume that the mask token is the last one. Therefore, Q_τ indicates that each token, except for the mask, remains unchanged with probability $1-\tau$ and transitions to the mask with probability τ. We then define the transition probability as:

$$p(x_t|x_{t+\Delta t}) = f(x_{t+\Delta t})Q_{\frac{\Delta t}{t+\Delta t}} \tag{2}$$

Thus, the interpolation is given by:

$$p(x_t|x_1) = f(x_1)Q_{1-t} = \text{Cat}[tf(x_1) + (1-t)f(M)] \tag{3}$$

where "Cat" denotes the categorical distribution.

3 Sampling of Flow

During the inference stage, CoFlow takes $\boldsymbol{x}_t$ and t as input and outputs the probabilistic distribution of $\boldsymbol{x}_1$. The model then computes the distribution of $\boldsymbol{x}_{t+\Delta t}$. For simplicity, we adopt residue-level notation x in the derivation. The distribution of $x_{t+\Delta t}$ can then be expressed using Bayes' rule as:

$$p(x_{t+\Delta t}|x_t;\theta) = \frac{p(x_t|x_{t+\Delta t})p(x_{t+\Delta t};\theta)}{p(x_t;\theta)} \tag{4}$$

Acoording to Equation 2&3, we can write $p(x_{t+\Delta t}|x_t;\theta)$ as:

$$p(x_{t+\Delta t}|x_t;\theta) = \frac{f(x_t)Q^\intercal_{\frac{t}{t+\Delta t}} \odot f_\theta Q_{1-t-\Delta t}}{f_\theta Q_{1-t} f^\intercal(x_t)} \tag{5}$$

where $\odot$ indicates Hardamard product. Consider Equation 1, we can write $f(x_t)Q^{\intercal}_{\frac{\Delta t}{t+\Delta t}}$ in Equation 5 as:

$$
\begin{aligned}
f(x_t)Q^{\intercal}_{\frac{\Delta t}{t+\Delta t}} &= f(x_t)\begin{bmatrix} \frac{t}{t+\Delta t} & & & \\ & \frac{t}{t+\Delta t} & & \\ & & \ddots & \\ \frac{\Delta t}{t+\Delta t} & \frac{\Delta t}{t+\Delta t} & \cdots & 1 \end{bmatrix} \\
&= \frac{\Delta t}{t+\Delta t}\delta(x_t, M) + \frac{t}{t+\Delta t}f(x_t)
\end{aligned} \tag{6}
$$

where δ indicates Kronecker Delta which is 1 when $x_t = M$ and is otherwise 0, and M indicates the mask token. The term $f_\theta Q_{1-t-\Delta t}$ is:

$$
\begin{aligned}
f_\theta Q_{1-t-\Delta t} &= f_\theta \begin{bmatrix} t+\Delta t & & & 1-t-\Delta t \\ & t+\Delta t & & 1-t-\Delta t \\ & & \ddots & \vdots \\ & & & 1 \end{bmatrix} \\
&= (t+\Delta t)f_\theta + (1-t-\Delta t)f(M)
\end{aligned} \tag{7}
$$

The last term $f_\theta Q_{1-t}f^{\intercal}(x_t)$ is:

$$
f_\theta Q_{1-t}f^{\intercal}(x_t) = (tf_\theta + (1-t)f(M))f^{\intercal}(x_t) \tag{8}
$$

Substitute Equation 6-8 into Equation 5, we can get if $x_t = M$:

$$
p(x_{t+\Delta t}|x_t;\theta) = \frac{\Delta t}{1-t}f_\theta + (1 - \frac{\Delta t}{1-t})f(M) \tag{9}
$$

otherwise $p(x_{t+\Delta t}|x_t;\theta) = 1$. Overall, this implies that if x_t is the mask token, at time step $t + \Delta t$, the state has a probability of $\frac{\Delta t}{1-t}$ to sample from the predicted distribution generated by the model, and a probability of $1 - \frac{\Delta t}{1-t}$ to remain as the mask token. However, if x_t is not the mask token, it will remain unchanged in the subsequent sampling step.

4 Model Training

CoFlow is initialized with ESM3 parameters and trained in two stages. In the first stage, MGnify30 (Lin et al., 2023b) is used as the training corpus, comprising 16 million proteins with sequence similarity below 30% and predicted structure metrics of pTM and pLDDT greater than 0.7. CoFlow is trained using the AdamW optimizer (Loshchilov and Hutter, 2019) with hyperparameters $\beta_1 = 0.9$, $\beta_2 = 0.98$, and a constant learning rate of 1×10^{-5}. The training process is conducted over 1 million steps on 8 Nvidia A800 GPUs. To enhance CoFlow's ability to generate structures closer to natural ones, the model is fine-tuned on the PDB dataset curated by (Campbell et al., 2024), which contains 23K proteins. During fine-tuning, the hyperparameters remain consistent with the first stage, except for a reduced learning rate of 3×10^{-6} and a shorter training duration of 10K steps. In the inference stage, proteins are sampled over 400 iterative steps, with sequence and structure tokens sampled alternately at each step.

5 Sampling Strategy

Since CoFlow is a joint generative model, sampling sequence and structure are flexible. Considering the generation order, we devise four sampling strategies, as outlined in Algorithm 1. For the "sequence first" strategy, we sample the sequence tokens $\boldsymbol{s}$ from time step 0 to 1, with the structure tokens $\boldsymbol{r}$ masked. Once the sequence sampling is completed, the structure is sampled from time step 0 to 1, while the input sequence tokens remain fixed. The "structure first" strategy follows a similar approach but with the order reversed. In both strategies, the modality generated later can fully capture the features of the former, but the former cannot capture the later. To address this, we propose two additional strategies. The "synchronous" strategy samples sequence and structure tokens simultaneously at each timestep, offering higher sampling efficiency since both modalities are sampled in parallel. The "asynchronous" strategy adopts a more fine-grained approach: in each timestep, the sequence is sampled first, and the sampled sequence is then input into the model for structure sampling in the subsequent step.

Algorithm 1 Four Sampling Strategies of CoFlow

```
procedure SEQUENCE FIRST                                        ▷ Strategy 1
    for t from 0 to 1 with step Δt do
        Sample s_{t+Δt} from CoFlow(s_t, r_0, t)
    end for
    for t from 0 to 1 with step Δt do
        Sample r_{t+Δt} from CoFlow(s_1, r_t, t)
    end for
end procedure

procedure STRUCTURE FIRST                                       ▷ Strategy 2
    for t from 0 to 1 with step Δt do
        Sample r_{t+Δt} from CoFlow(s_0, r_t, t)
    end for
    for t from 0 to 1 with step Δt do
        Sample s_{t+Δt} from CoFlow(s_t, r_1, t)
    end for
end procedure

procedure SYNCHRONOUS                                           ▷ Strategy 3
    for t from 0 to 1 with step Δt do
        Sample r_{t+Δt} and s_{t+Δt} from CoFlow(s_t, r_t, t)
    end for
end procedure

procedure ASYNCHRONOUS                                          ▷ Strategy 4
    for t from 0 to 1 with step Δt do
        Sample r_{t+Δt} from CoFlow(s_t, r_t, t)
        Sample s_{t+Δt} from CoFlow(s_{t+Δt}, r_t, t)
    end for
end procedure
```

In evaluation experiments, to account for the generation order and capacity to model and generate secondary structures, four distinct sampling strategies for ESM3 are implemented:

- Sequence→structure ($\boldsymbol{s} \to \boldsymbol{r}$): generating sequence first followed by structure;
- Structure→sequence ($\boldsymbol{r} \to \boldsymbol{s}$): generating structure first followed by sequence;
- Secondary Structure → Sequence → Structure ($ss \to \boldsymbol{s} \to \boldsymbol{r}$): generating secondary structure first, then sequence and tertiary structure;
- Secondary Structure → Structure → Sequence ($ss \to \boldsymbol{r} \to \boldsymbol{s}$): generating secondary structure first, then tertiary structure and sequence.

6 Evaluation of Structure VQ-VAE

The VQ-VAE is composed of an encoder providing a rich, learned representation of the local neighborhood for each residue and associating it with one of 4,096 structure tokens (+4 special tokens), and a corresponding decoder to enable decoding of discrete structure tokens back to three-dimensional atomic coordinates. As the CoFlow runs at the token level, the encoding and decoding accuracy of the structure VQ-VAE is critical for the success of backbone design. The structure representations generated by the VQ-VAE exhibit strong separability in terms of structure classification (Fig.S3a), indicating that the encoder effectively captures the spatial features of the protein structure. The decoder has demonstrated the ability to accurately recover the structure (Fig.S3b), with most proteins exhibiting a backbone (atoms including N, C_α, C) root-mean-square deviation (RMSD) of less than 2Å, except for those with sparse tertiary contacts, disordered regions, or unresolved coordinates. Moreover, the decoder shows robust performance in the presence of noise (Fig.S3c). When the replacement tokens are chosen from the top 10 nearest neighbors, even replacing all structure tokens results in an average backbone RMSD below 1Å. When replacement tokens are randomly selected from the top 40 neighbors, the backbone RMSD increases but remains below 2.5Å. These findings demonstrate that the VQ-VAE effectively encodes three-dimensional protein structures into a discrete space with high separability and can reliably reconstruct the original structure from these tokens, thereby enabling the development of generative models in this discrete structural space.

References

Josh Abramson, Jonas Adler, Jack Dunger, Richard Evans, Tim Green, Alexander Pritzel, Olaf Ronneberger, Lindsay Willmore, Andrew J Ballard, Joshua Bambrick, et al. Accurate structure prediction of biomolecular interactions with alphafold 3. *Nature*, pages 1–3, 2024.

Frances H Arnold. Directed evolution: creating biocatalysts for the future. *Chemical engineering science*, 51(23):5091–5102, 1996.

Nadav Brandes, Dan Ofer, Yam Peleg, Nadav Rappoport, and Michal Linial. Proteinbert: a universal deep-learning model of protein sequence and function. *Bioinformatics*, 38 (8):2102–2110, 2022.

Tom Brown, Benjamin Mann, Nick Ryder, Melanie Subbiah, Jared D Kaplan, Prafulla Dhariwal, Arvind Neelakantan, Pranav Shyam, Girish Sastry, Amanda Askell, et al. Language models are few-shot learners. In *NeurIPS*, volume 33, pages 1877–1901, 2020.

Andrew Campbell, Jason Yim, Regina Barzilay, Tom Rainforth, and Tommi Jaakkola. Generative flows on discrete state-spaces: Enabling multimodal flows with applications to protein co-design. In *Proceedings of the 41st ICML*, PMLR, 2024.

Peng Cheng, Cong Mao, Jin Tang, Sen Yang, Yu Cheng, Wuke Wang, Qiuxi Gu, Wei Han, Hao Chen, Sihan Li, et al. Zero-shot prediction of mutation effects with multimodal deep representation learning guides protein engineering. *Cell Research*, 34(9):630–647, 2024.

Alexander E Chu, Jinho Kim, Lucy Cheng, Gina El Nesr, Minkai Xu, Richard W Shuai, and Po-Ssu Huang. An all-atom protein generative model. *Proceedings of the National Academy of Sciences*, 121(27):e2311500121, 2024a.

Alexander E Chu, Jinho Kim, Lucy Cheng, Gina El Nesr, Minkai Xu, Richard W Shuai, and Po-Ssu Huang. An all-atom protein generative model. *Proceedings of the National Academy of Sciences*, 121(27):e2311500121, 2024b.

Ahmed Elnaggar, Michael Heinzinger, Christian Dallago, Ghalia Rehawi, Yu Wang, Llion Jones, Tom Gibbs, Tamas Feher, Christoph Angerer, Martin Steinegger, et al. Prottrans: Toward understanding the language of life through self-supervised learning. *IEEE transactions on pattern analysis and machine intelligence*, 44(10):7112–7127, 2021.

Noelia Ferruz, Steffen Schmidt, and Birte Höcker. Protgpt2 is a deep unsupervised language model for protein design. *Nature communications*, 13(1):4348, 2022.

Thomas Hayes, Roshan Rao, Halil Akin, Nicholas J Sofroniew, Deniz Oktay, Zeming Lin, Robert Verkuil, Vincent Q Tran, Jonathan Deaton, Marius Wiggert, et al. Simulating 500 million years of evolution with a language model. *Science*, 387(6736):850–858, 2025.

Jonathan Ho, Ajay Jain, and Pieter Abbeel. Denoising diffusion probabilistic models. *NeurIPS*, 33:6840–6851, 2020.

John B Ingraham, Max Baranov, Zak Costello, Karl W Barber, Wujie Wang, Ahmed Ismail, Vincent Frappier, Dana M Lord, Christopher Ng-Thow-Hing, Erik R Van Vlack, et al. Illuminating protein space with a programmable generative model. *Nature*, 623 (7989):1070–1078, 2023.

John Jumper, Richard Evans, Alexander Pritzel, Tim Green, Michael Figurnov, Olaf Ronneberger, Kathryn Tunyasuvunakool, Russ Bates, Augustin Žídek, Anna Potapenko, et al. Highly accurate protein structure prediction with alphafold. *nature*, 596(7873): 583–589, 2021.

Jacob Devlin Ming-Wei Chang Kenton and Lee Kristina Toutanova. Bert: Pre-training of deep bidirectional transformers for language understanding. In *Proceedings of naacL-HLT*, volume 1, page 2. Minneapolis, Minnesota, 2019.

Julia Koehler Leman, Brian D Weitzner, Steven M Lewis, Jared Adolf-Bryfogle, Nawsad Alam, Rebecca F Alford, Melanie Aprahamian, David Baker, Kyle A Barlow, Patrick Barth, et al. Macromolecular modeling and design in rosetta: recent methods and frameworks. *Nature methods*, 17(7):665–680, 2020.

Zeming Lin, Halil Akin, Roshan Rao, Brian Hie, Zhongkai Zhu, Wenting Lu, Nikita Smetanin, Robert Verkuil, Ori Kabeli, Yaniv Shmueli, et al. Evolutionary-scale prediction of atomic-level protein structure with a language model. *Science*, 379(6637): 1123–1130, 2023a.

Zeming Lin, Halil Akin, Roshan Rao, Brian Hie, Zhongkai Zhu, Wenting Lu, Nikita Smetanin, Robert Verkuil, Ori Kabeli, Yaniv Shmueli, et al. Evolutionary-scale prediction of atomic-level protein structure with a language model. *Science*, 379(6637): 1123–1130, 2023b.

Sidney Lyayuga Lisanza, Jacob Merle Gershon, Samuel WK Tipps, Jeremiah Nelson Sims, Lucas Arnoldt, Samuel J Hendel, Miriam K Simma, Ge Liu, Muna Yase, Hongwei Wu, et al. Multistate and functional protein design using rosettafold sequence space diffusion. *Nature biotechnology*, pages 1–11, 2024.

Yufeng Liu, Sheng Wang, Jixin Dong, Linghui Chen, Xinyu Wang, Lei Wang, Fudong Li, Chenchen Wang, Jiahai Zhang, Yuzhu Wang, et al. De novo protein design with a denoising diffusion network independent of pretrained structure prediction models. *Nature Methods*, pages 1–10, 2024.

Ilya Loshchilov and Frank Hutter. Decoupled weight decay regularization. In *ICLR*, 2019.

Ali Madani, Ben Krause, Eric R Greene, Subu Subramanian, Benjamin P Mohr, James M Holton, Jose Luis Olmos, Caiming Xiong, Zachary Z Sun, Richard Socher, et al. Large language models generate functional protein sequences across diverse families. *Nature Biotechnology*, 41(8):1099–1106, 2023.

Igor Melnyk, Vijil Chenthamarakshan, Pin-Yu Chen, Payel Das, Amit Dhurandhar, Inkit Padhi, and Devleena Das. Reprogramming pretrained language models for antibody sequence infilling. In *ICML*, pages 24398–24419. PMLR, 2023.

Ananthan Nambiar, Maeve Heflin, Simon Liu, Sergei Maslov, Mark Hopkins, and Anna Ritz. Transforming the language of life: transformer neural networks for protein prediction tasks. In *Proceedings of the 11th ACM international conference on bioinformatics, computational biology and health informatics*, pages 1–8, 2020.

Aditya Ramesh, Mikhail Pavlov, Gabriel Goh, Scott Gray, Chelsea Voss, Alec Radford, Mark Chen, and Ilya Sutskever. Zero-shot text-to-image generation. In *ICML*, pages 8821–8831. Pmlr, 2021.

Milong Ren, Tian Zhu, and Haicang Zhang. Carbonnovo: Joint design of protein structure and sequence using a unified energy-based model. In *41st ICML*.

Robin Rombach, Andreas Blattmann, Dominik Lorenz, Patrick Esser, and Björn Ommer. High-resolution image synthesis with latent diffusion models. In *Proceedings of the IEEE/CVF conference on computer vision and pattern recognition*, pages 10684–10695, 2022.

Chentong Wang, Yannan Qu, Zhangzhi Peng, Yukai Wang, Hongli Zhu, Dachuan Chen, and Longxing Cao. Proteus: Exploring protein structure generation for enhanced designability and efficiency. In *Proceedings of the 41st ICML*, volume 235 of *PMLR*, pages 51376–51395. PMLR, 21–27 Jul 2024.

Lihao Wang, Yuning Shen, Yiqun Wang, Huizhuo Yuan, Yue Wu, Quanquan Gu, et al. Protein conformation generation via force-guided se (3) diffusion models. In *41st ICML*.

Joseph L Watson, David Juergens, Nathaniel R Bennett, Brian L Trippe, Jason Yim, Helen E Eisenach, Woody Ahern, Andrew J Borst, Robert J Ragotte, Lukas F Milles, et al. De novo design of protein structure and function with rfdiffusion. *Nature*, 620 (7976):1089–1100, 2023.

Kevin E Wu, Kevin K Yang, Rianne van den Berg, Sarah Alamdari, James Y Zou, Alex X Lu, and Ava P Amini. Protein structure generation via folding diffusion. *Nature communications*, 15(1):1059, 2024.

Jason Yim, Brian L. Trippe, Valentin De Bortoli, Emile Mathieu, Arnaud Doucet, Regina Barzilay, and Tommi Jaakkola. Se(3) diffusion model with application to protein backbone generation. In *Proceedings of the 40th ICML*, PMLR. PMLR, 2023.

7 Supplemental Table and Figures

Table S1: Glossary of Acronyms

**Acronym**	**Definition**
RMSD	Root-Mean-Square Deviation
PDB	Protein Data Bank
VQ-VAE	Vector Quantised - Variational AutoEncoder
pTM	predicted Template Modeling score
pLDDT	predicted Local Distance Difference Test
SE(3)	Special Euclidean Group in 3 dimensions
NSR	Native Sequence Recovery

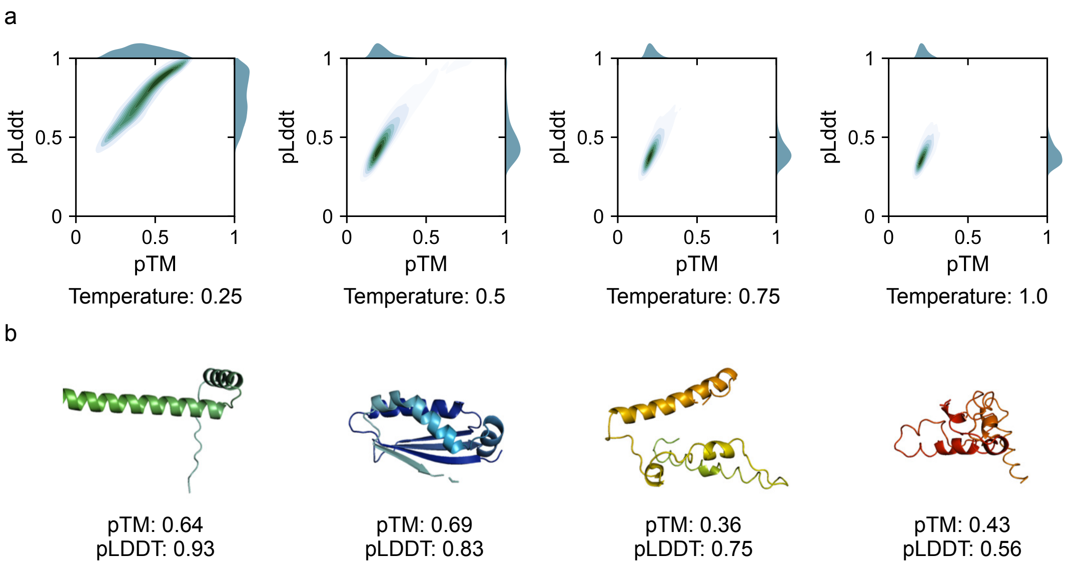

Figure S1: Evaluation of ESM3-open on Protein Backbone Design. We evaluate the unconditional backbone design capabilities of ESM3-open at four different temperatures: 0.25, 0.5, 0.75, and 1.0. ESM3-open is required to generate 2,000 protein backbones of random lengths from scratch. It takes masked tokens as input and sequentially unmasks them based on the entropy of the logits in steps of one-eighth of the protein length. The structure VQ-VAE decoder is used to convert the generated structure tokens into three-dimensional coordinates of the backbone atoms, yielding both pTM and pLddt scores. (a) Joint distribution of pTM and pLddt scores for the generated backbones across the four sampling temperatures. (b) Visualization of randomly generated protein backbones by ESM3-open.

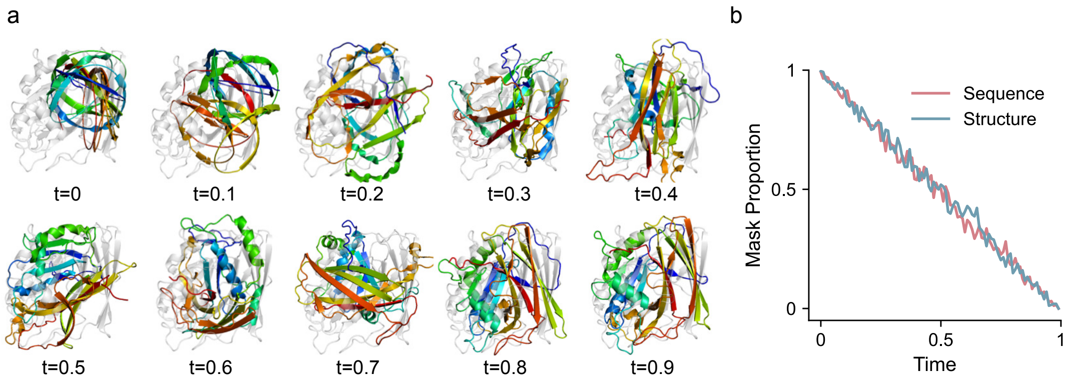

Figure S2: Visualization of Structure Interpolation of CoFlow. (a) Structure interpolation from time 0 to 0.9. The grey background represents the original structure, while the colored structure corresponds to the structure reconstructed from interpolated tokens. (b) The proportion of masked sequence and structure tokens as a function of time.

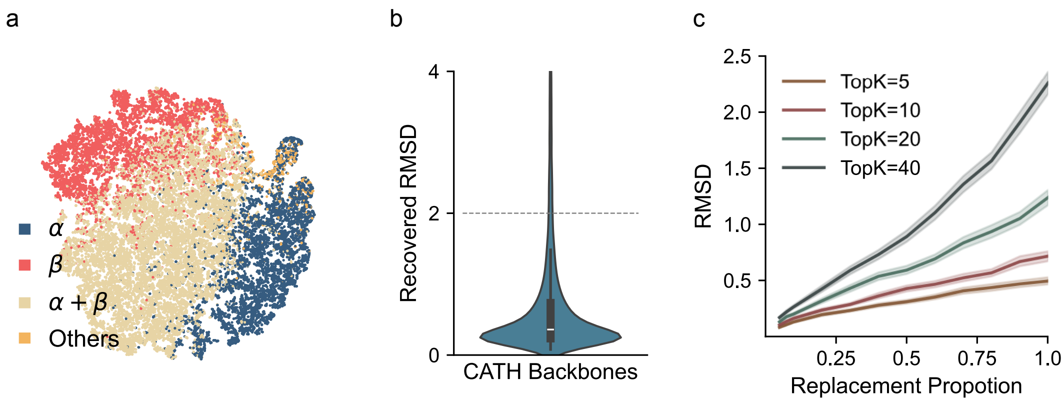

Figure S3: Evaluation of Structure VQ-VAE. (a) Visualization of the encoded representations of protein structures from the CATH dataset, which classifies proteins into four categories: mainly alpha helix (α), mainly beta sheet (β), alpha+beta ($\alpha+\beta$), and others (including those with few secondary structures or special cases). The protein representations are obtained by averaging the residue representations from the VQ-VAE encoder, followed by dimensionality reduction using t-SNE. (b) Distribution of RMSD between the original CATH structures and the structures recovered by VQ-VAE. (c) RMSD values between the original CATH structures and the corresponding recovered structures from noised structure tokens. To evaluate the robustness of the VQ-VAE decoder, we randomly replace structure tokens with their nearest neighbors in vector space. Specifically, each token is replaced by one randomly selected from its top-5, top-10, top-20, or top-40 nearest neighbors, and the decoder is then used to reconstruct the structure from these noised tokens.

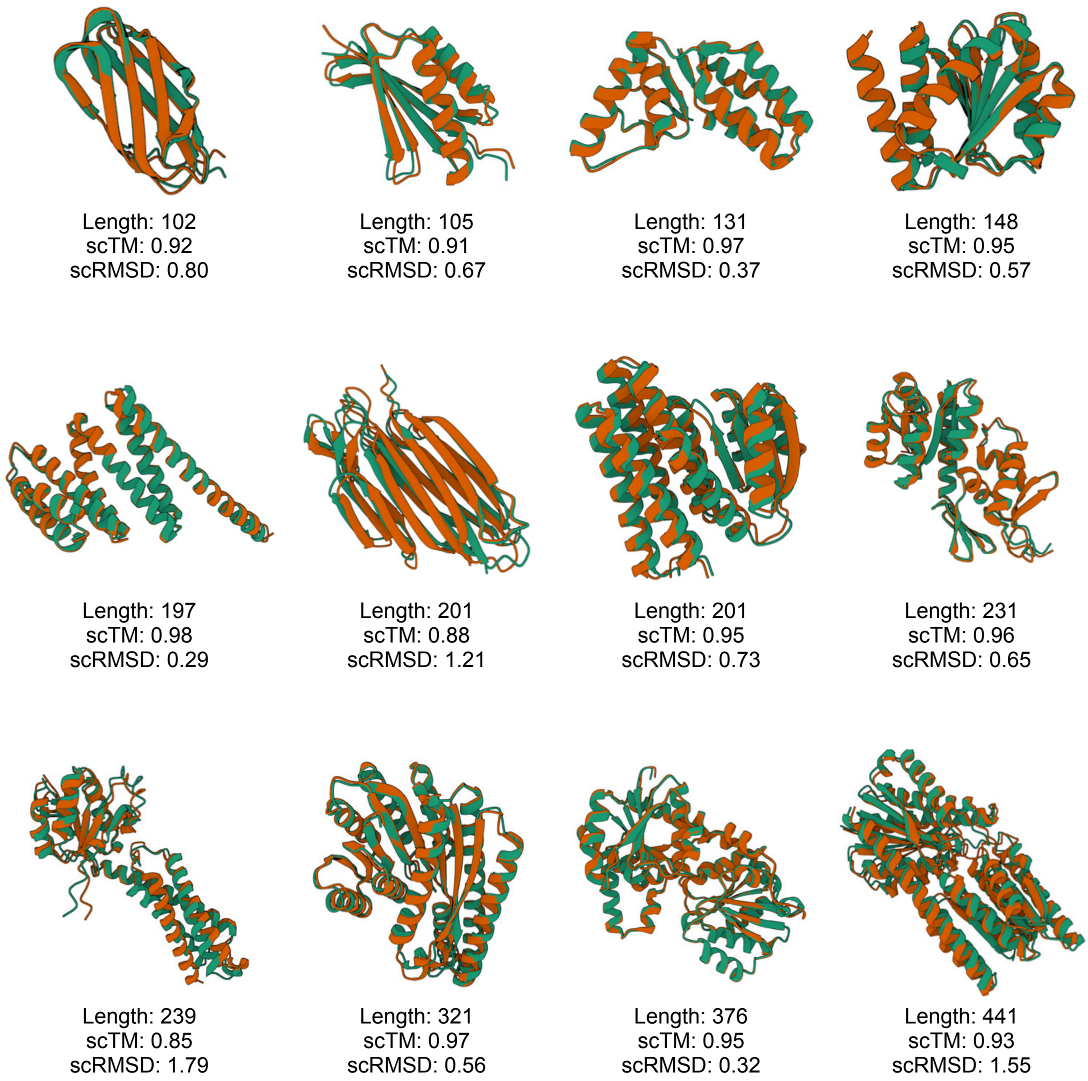

Figure S4: Unconditional Generation Instances. The green color represents the structures generated by CoFlow, while the orange color represents the predicted structures.

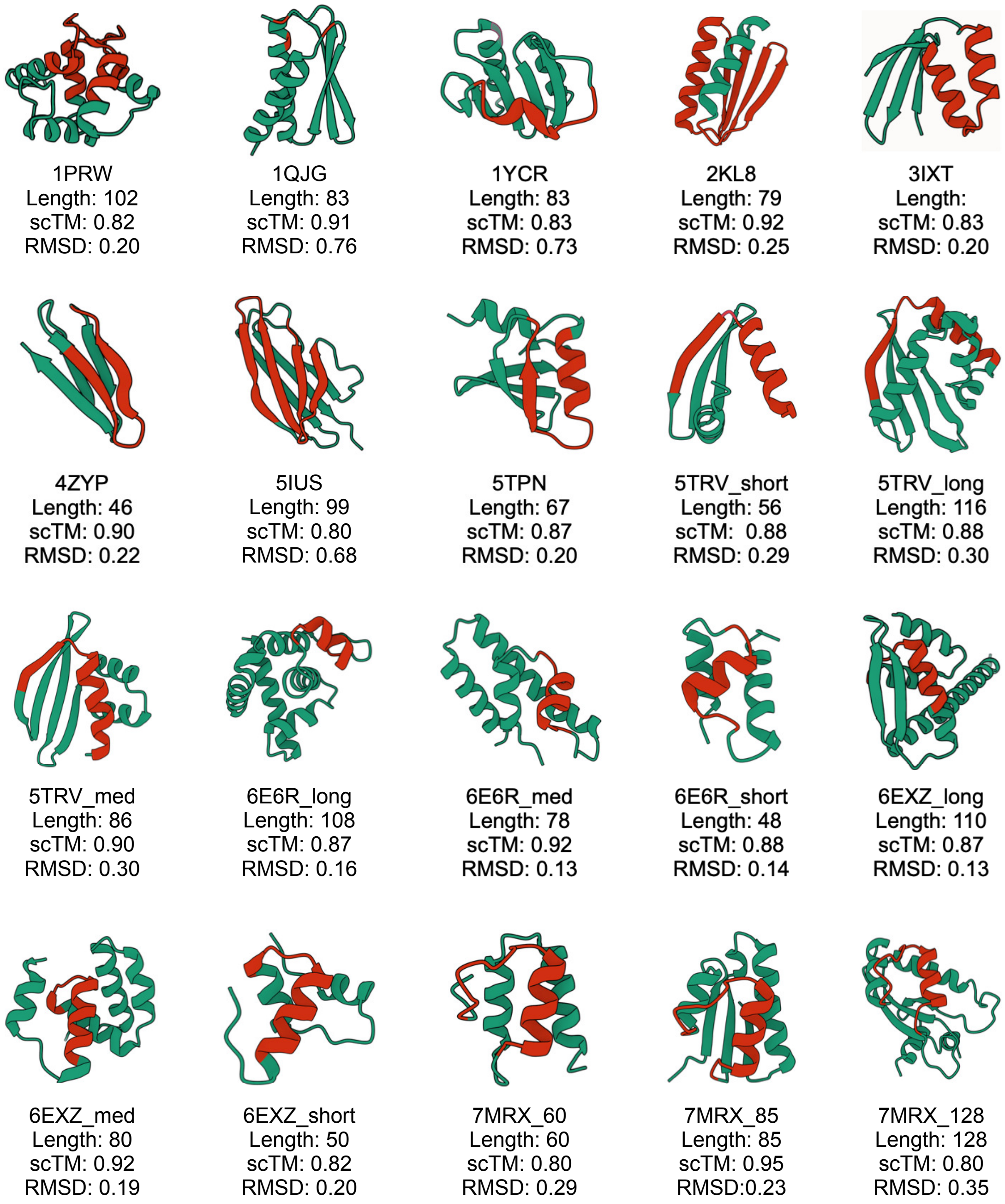

Figure S5: Successful Instances Designed by CoFlow. The red color represents the given motif, while the teal color indicates the generated part.

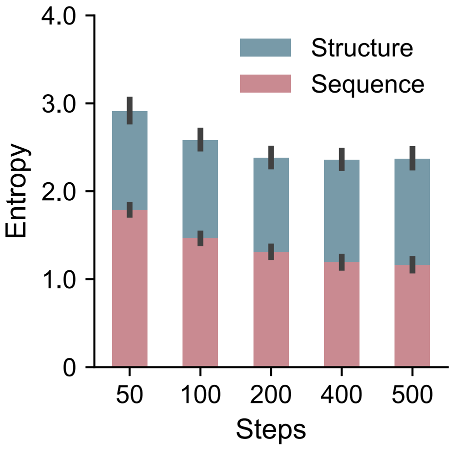

Figure S6: Generation entropy of ESM3 with difference sampling steps.
